# Towards Memory-Aware Services and Browsing through Lifelogging Sensing

**DOI:** 10.3390/s131115113

**Published:** 2013-11-05

**Authors:** Lorena Arcega, Jaime Font, Carlos Cetina

**Affiliations:** School of Computer Science, San Jorge University, Zaragoza 50830, Spain; E-Mails: larcega@usj.es (L.A.); jfont@usj.es (J.F.)

**Keywords:** wearable computing, lifelogging sensors, internet of things

## Abstract

Every day we receive lots of information through our senses that is lost forever, because it lacked the strength or the repetition needed to generate a lasting memory. Combining the emerging Internet of Things and lifelogging sensors, we believe it is possible to build up a Digital Memory (Dig-Mem) in order to complement the fallible memory of people. This work shows how to realize the Dig-Mem in terms of interactions, affinities, activities, goals and protocols. We also complement this Dig-Mem with memory-aware services and a Dig-Mem browser. Furthermore, we propose a RFID Tag-Sharing technique to speed up the adoption of Dig-Mem. Experimentation reveals an improvement of the user understanding of Dig-Mem as time passes, compared to natural memories where the level of detail decreases over time.

## 1. Introduction

Memory is the ability of the brain to store information. Given enough stimuli and rehearsal, we can remember data for many years and recall that information whenever we need it. However, not all stimuli are strong enough to generate a memory that can be recalled later. Every day we receive a lot of information through our senses that is lost forever, because it lacked the strength or the repetition needed to generate a lasting memory.

In many situations, that information can be important or necessary: where did I put my keys, what was the name of that man I just met, do I need to buy more milk, *etc.* As time passes, the aging brain loses information, or is not able anymore to recall some memories. In the extreme situation of Alzheimer's disease, this loss will have devastating consequences for everyday life.

The use of external memory aids to help people to compensate for their memory's deficits is thought to be one of the most valuable and effective ways to reduce the impact of Age-related Memory Impairment (AMI) on everyday functioning [1]. The vision of lifelogging technologies [2] can significantly contribute to realize these external memory aids. Lifelogging technologies sense multiple kinds of data as complete and automatically as possible for storing collections of heterogeneous digital objects that users generate or encounter, including photos, email, web pages and sounds. Although the details are different, overall, these lifelogging approaches propose wearable systems mainly based on still cameras (head-mounted or worn around the neck) [3] or still cameras complemented with sensors such as sound devices [4] or biosensor readings [5]. However, recent works have found that users with collections of thousands of digital photos never access the majority of them [6,7]. These works raise questions about the utility of a digital archive in remembering or reviewing the past.

We believe that the combination of current mobile sensors (such as GPS or RFID readers) and activity recognition techniques [8–11] can enable us to build up a Digital Memory (hereafter Dig-Mem). Whenever a person touches any digitally identified thing, its unique identification and the time and location of the interaction is stored in some persistent digital storage, the Dig-Mem of this person. Furthermore, Dig-Mem structures these interactions into activities, determining if they follow a specific protocol or accomplish a goal. For example, when the user touches a cup, the carton of milk, a spoon and the coffee box in her kitchen in the morning, our approach structures this information as Having Breakfast activity. The recognized activities can be use to trigger autobiographical memory [12] about past events. That is, Dig-Mem could provide the cues to remember past events by means of the associative thinking of the human mind [13].

With Dig-Mem we focus on the part of the natural memory known as declarative memory [14]. Declarative memory (or explicit) is one of two types of long term human memory. It refers to memories which can be consciously recalled such as facts and events. Its counterpart is known as procedural memory (or implicit), which refers to unconscious memories such as skills (e.g., learning to ride a bicycle).

A prerequisite for the realization of the Dig-Mem is the existence of Digital Object Identifiers (DOIs) in the world, as proposed by the Internet of Things paradigm [15,16]. An increasing number of everyday objects (clothing, appliances, books, *etc.*) are being electronically identified using RFID or NFC tags. This suggests that the DOIs existence prerequisite will be fulfilled in the near future. We expect this issue to fade, but meanwhile, early users have to put the RFID tags themselves. Our proposal of a Dig-Mem will use these DOIs to store information about the interaction with an increasing number of objects, tagged not only by active users of the Internet of Things, but also by manufacturers and vendors, and even by non technical-savvy customers.

The contribution of this work is two-fold: (1) memory-aware services that can be used to remember interactions of persons with things (even when the things are not physically present). These services have been implemented and validated in real environments by students, reaching interesting conclusions about the improvement of catalogs of memory-aware services; (2) A Dig-Mem Browser capable of navigating through those digital memories and represent them thanks to a organization of that memories into activities done with that things, protocols followed using that things and goals accomplished with the manipulation of them. By means of the browser we have performed a case study comparing the evolution of natural memory and Dig-Mem over time. Evidences support that Dig-Mem evolves with time getting close to the user understanding and may exist some kind of synergy between Dig-Mem and natural memory.

From this point, Section 2 presents the overview of our work (memory expressiveness and population techniques). Section 3 presents affinities between interactions. Section 4 describes the aggregation of interactions into activities. Section 5 presents The Memory-Aware Services. Section 6 describes the browser to navigate through Dig-Mem. Section 7 describes the evaluation carried out to validate this work. Section 8 presents a comparison between Natural and Dig-Mem. Section 9 discusses related work. In the final section, we make some conclusions.

## 2. Overview of the Approach

This section provides a brief overview of the main concepts of the paper that are going to be developed throughout the next sections.

**Figure 1.**
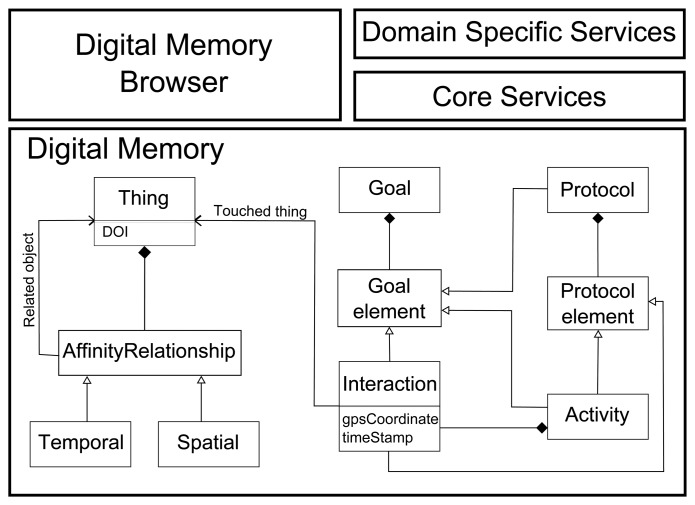
Dig-Mem Overview. Main elements that can be mounted on our bases of Dig-Mem (top). The core element of our approach, Digital Memory concepts (bottom).

The core element of our approach, as seen at the bottom of Figure 1, is the Digital Memory concepts. Dig-Mem stores structured information about the *interactions* of persons with tagged things, and *affinities* between objects calculated from the interactions. These affinities represent relationships between the objects in the environment. The interactions are grouped into activities, following some rules defined by the user. A user can also define goals or protocols over the activities and interactions.

For example, we can keep track of all the times that the user opens or closes the door of her car (interactions). And we can detect that the user touches the key and the door of her car in the same spatio-temporal frame (affinity). But we can also detect the activity of starting driving the car (interaction with the door followed by interaction with the keys and the handbrake), or even we can check for compliance with the protocol of not drinking before driving.

*Core services* can be offered to use the information stored on Dig-Mem. These services provide basic information about Dig-Mem elements, as interactions with a specific object, detected relationships with other things or activities where a specific object is involved.

Several core services can be combined or tailored together to provide solutions to domain specific problems or needs. These *Domain Specific Services* typically are triggered with the user touching a thing of interest and are capable of navigating the Dig-Mem to offer information specific to the domain. For example, by touching the key of the car, the user can ask for the parking location. And holding the hotel key card, the user can ask for a route to the hotel.

Lastly, a *Dig-Mem Browser* graphically represents the information stored in the Dig-Mem allowing for direct exploration of Dig-Mem in those situations when access to the interactions and affinities stored within are needed.

### 2.1. Dig-Mem Expressiveness

The expressiveness of Dig-Mem enables the characterization of the interactions between the user and things. To decide the dimensions included in Dig-Mem we have taken into account the increasing popularity of smartphones and their capabilities. Modern smartphones incorporate many sensors that can be used to capture information. With this in mind, we have selected the following basic dimensions: *thing* (DOI measured using RFID or NFC tag readers), *time* (measured with the reader or the smartphone clock), and *location* (using GPS positioning services).

For localization we use the GPS of the mobile phone, and we are aware of the limitations of GPS. We expect this issue to fade as sensors improve, but it is a significant concern for any deployment on currently available smartphone hardware. To address these limitations we differentiate two situations, indoors-location and outdoors-location. We have decided to provide a system for exploration rather than navigation, which has proven successful results in other works [8–10].

For indoor environments we have two types of RFID tag, static (*i.e.*, bathroom faucet) and nomadic (*i.e.*, wallet) tags, to alleviate this issue. RFID tags allow us to save additional information on it. So, we recorded static labels with their location. Thus, when the user touches the bathroom faucet the system will save the location of the interaction as “Bathroom”. Water and metal absorb the radio waves that most RFID tags use; but more careful tag positioning, and using newer RFID tags that are optimized for placement near liquids and metal, could mitigate this.

When our approach is used outside a familiar environment, such as the home, we obtain the geocoding with the Google API to find out the place and address where the user is. Google API enables us to get the points of interest (POIs) that the user has visited. We do this using the technique used by [8], they compare the POI address with the closest address from the user's position. This allows us to not only show geometric coordinates or an address, but show the name of the point of interest. This technique enables us to save substantial changes in the location of the user. Although we cannot overcome the sensor limitations entirely, we are designing the system to be as robust as possible to sensor errors.

### 2.2. Techniques for Populating the Dig-Mem

The Dig-Mem of a person is the electronic storage of the DOIs associated with objects with which the person has come into contact, together with some additional information. Following previous work in the field [17], one RFID or NFC tag is attached to each object in the real world. To read the identification number stored in the tag we will use a tag reader, that will send the information to the Dig-Mem.

**Figure 2.**
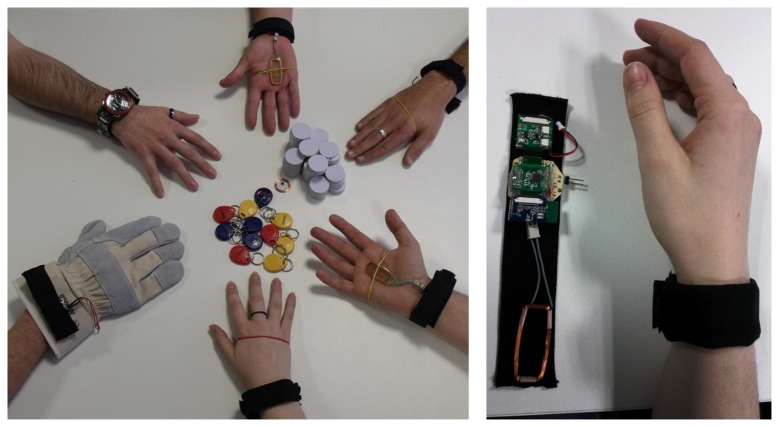
Different prototypes of our RFID Tag reader, such as a glove, a bracelet and a watch (**left**). The final prototype used in our evaluations (**right**).

Two settings have been explored for the population of the Dig-Mem: *foreground feed* (active) and *background feed* (passive):

1. To support foreground feed we have used a smartphone with an out-of-the-box NFC tag reader (Nexus S). To populate its Dig-Mem users must consciously touch things with the smartphone.
2. To support background feed we have used a RFID tag reader attached to a Bluetooth-capable wristwatch (see Figure 2). The range of the RFID reader has been tuned to include only the hand where the watch is worn. Therefore, the user feeds the Dig-Mem whenever he touches a tagged thing, consciously or not.

In foreground feed, the objects must be touched with the smartphone to be stored in Dig-Mem. In background feed, the range of the RFID antenna in the wristwatch is tuned so that every tagged object touched with that hand will be sent via Bluetooth and stored automatically in Dig-Mem. The advantage of foreground feed is that there is no need for additional hardware, because the Dig-Mem persistent storage and the NFC/RFID tag reader are integrated in the same smartphone. The disadvantage is that the interaction must be actively initiated by the user, and so he must be aware of the existence of the Digital Memory and the need to populate it with information from the world. These characteristics are reversed in background feed, because we need additional hardware (our RFID/BT wristwatch) but there is no need for active participation of the user in the population of the Dig-Mem. With background feed the user populates his Dig-Mem contents in a more natural and unobtrusive way.

## 3. Affinity Relationships in the Dig-Mem

Interactions comprise the core of the Dig-Mem. But to offer core services, we need to detect the relationships between different things of interest in the environment of the user. Given some criteria that defines a relationship between objects, we will say that two things in our Dig-Mem have *affinity* if they verify those criteria.

Given the expressiveness of Dig-Mem described in Section 2.1, we can formulate affinity in the following terms:

- Two things have **spatial affinity** when they normally share the same location, even at different times. For example, the relationship that is established between a book and the bookshelf where it is stored.
- Two things have **temporal affinity** when they are touched within a given time interval. The span of this interval will be dependent on the application domain. For example, in the relationship between the credit card and the wallet there exists a *temporal affinity* because the user must touch the wallet just before and just after using the credit card.

### 3.1. Implementation Details of the Affinities

Each object of interest to the user must be supplied with an RFID tag attached to the position in the object where there are more possibilities to be touched, like the knob of a door, the handle of a pan or the key ring, because one of the aims of the work is to be as unobtrusive as possible. In this regard, under certain circumstances, it may be advisable that the user does not require to touch the object but rather to be close to it. This may depend on the type of object (*i.e.*, a painting). Thus, other implementations may be configured to be activated upon touching or in close proximity as well.

Accordingly, our implementation of portable reader for the Dig-Mem includes an RFID reader which is equipped with an antenna to increase the range of the interaction of the reader with the electromagnetic tag. RFID power and antenna size are calculated according to common knowledge to limit interactions with other tags in the proximity area of the portable reader. The identification code read by the RFID reader is transmitted via a low-power RF transmitter module using the standard communication protocol Bluetooth. The reader is powered by a small battery to make it portable and light-weight.

As the user interacts with the things of his environment, the portable reader begins to collect DOIs from electromagnetic tags. Many readings are possible for the same thing, if the user holds the object while moving around, or if portable reader is configured to read several times per minute. An Interaction Point is stored in the smart phone for each such reading, together with the time and location of the interaction, as provided by clock and GPS receiver.

**Figure 3.**
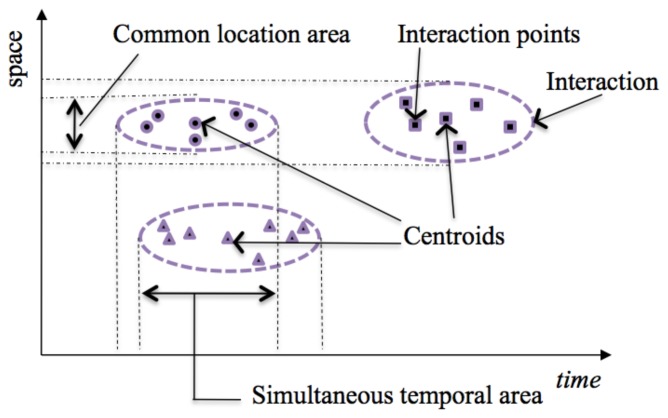
Graph with examples of information read or calculated by the system and stored in the smart phone. It represents an example of Interaction Points for three different Interactions, each one represented by a different shape.

Figure 3 shows a graph with time in the horizontal axis and space as a single dimension in the vertical axis with examples of information read or calculated by the system and stored in the smart phone. This figure represents an example of Interaction Points for three different Interactions, each one represented by a different shape. Interactions Points for the same thing grouped together can be thought as pertaining to a unique interaction of the person with the thing, with a temporal interval and a location area.

Figure 3 also represents the clusters of points identified by means of a statistical clustering algorithm based on distance between tag reading events. To do this, we use K-means method. This method aims to partition a set *n* into *k* clusters in which each observation belongs to the cluster closest to the average. The information supplied by this method is the centroid of the cluster, which is the Interaction Point representative of the Interaction, together with an estimation of the time interval and spatial boundary of the interaction.

Finally, the intersection of the temporal or spatial extensions of the cluster can lead to the establishment of affinities between different Interactions. The system can determine the degree of spatial affinity between two interactions based on the existence of a common location area for the clusters. To calculate the spatial span of the cluster, we use the location of the centroid plus the common location area (See Figure 3). The system can similarly determine the degree of temporal affinity between two interactions based on the existence of a simultaneous temporal interval for the clusters. To calculate the temporal span of the cluster, we use the temporal coordinate of the centroid plus the temporal extension of the cluster as calculated by clustering analysis.

Figure 3 shows an example of information related to affinities between two objects generated by affinity analysis and stored in the Dig-Mem. Together with the IDs of both things (ID-1 and ID-2), a frequency count is kept for the number of spatial affinities detected between those two same objects, and a frequency count is kept for the number of temporal affinities detected between those two same objects.

To limit the number of things with affinities shown in the memory-aware services, a threshold value is set for the frequency counts. By selecting a threshold value for the frequency count of the affinity between two objects, two type of affinities can be distinguished. Sparse affinity when frequency count is less than threshold value. Reinforced affinity when frequency count is greater than threshold value. There is usually a correspondence between a reinforced affinity between two DOIs and a relevant relationship between the two correspondent things when the user employs them, so services based on affinity can be configured to show only objects with reinforced affinity, limiting the number of objects shown to the user when asking for an affinity service.

In the example process described, we start touching an object to know its related objects. In addition, this process can begin searching by the object in the smartphone and navigating to its affinities obtaining the same result as before. However, touching an object to search their related objects doesn't disrupt the natural interaction between user and objects.

**Figure 4.**
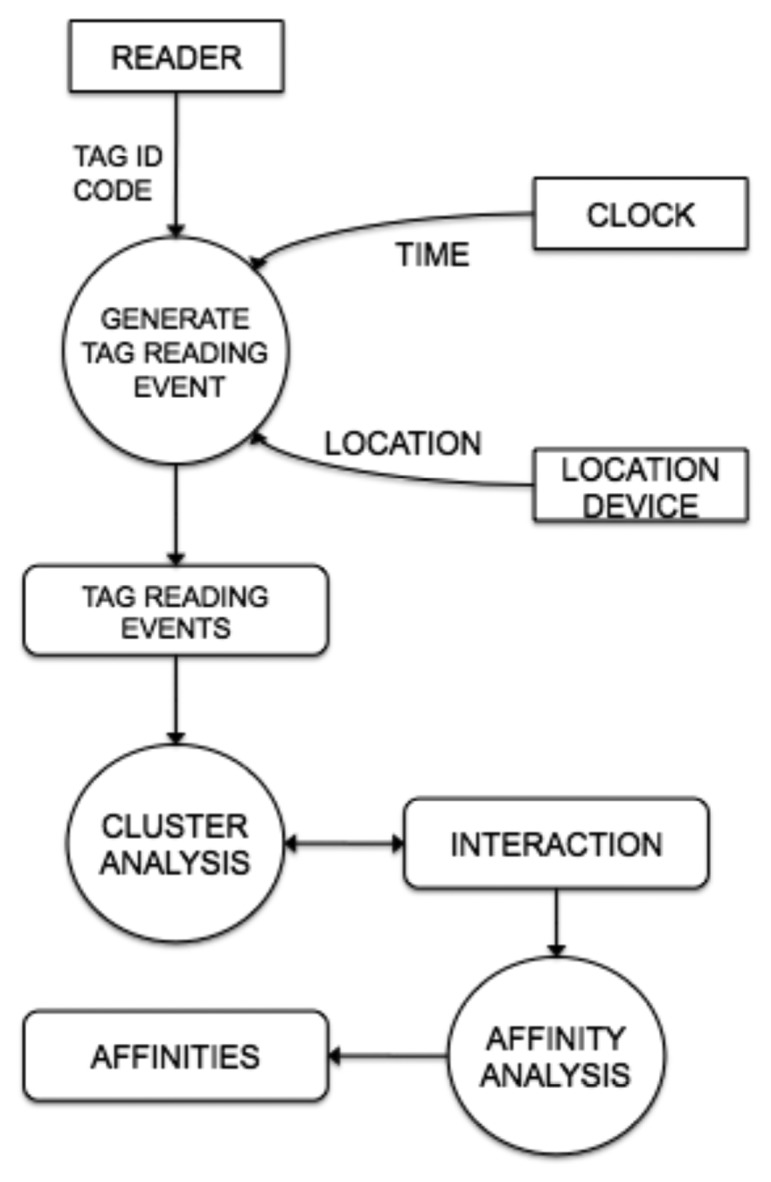
Steps performed by the system to generate interactions and affinities. In the firt step, the RFID-reader reads an electromagnetic tag and sends its DOI to the Generate Tag Reading Event step. The information is packaged as a Interaction Point. Step Cluster Analysis looks for an interaction where the Interaction Point is near to its centroid. Step Affinity Analysis reads new interactions and searches for affinities.

An example of the steps performed by the system to generate interactions and affinities is shown in Figure 4. The RFID-reader reads an electromagnetic tag and sends its DOI to the Generate Tag Reading Event step. This step obtains the time and location from the smart phone clock and GPS receiver and packs all these data as a Interaction Point that is stored in the smart phone. Step Cluster Analysis reads the new Interaction Point and looks for an interaction where the Interaction Point is near to its centroid, based on distance. If the interaction is found, its centroid, duration and spatial extension are updated. If no interaction is found, a new one is created with the Interaction Point as centroid and zero value for temporal and spatial extensions. Step Affinity Analysis reads new interactions and searches for affinities. If an existing affinity is found, its frequency counts are updated. If a new affinity is found, it is created with value one for its frequency counts, and stored in the smartphone.

The process followed by a person that wants to receive information about his interactions with a thing when he is not able to read the tag (for example his car) is described as follows. The process begins when the person uses the portable reader to touch a related object, for example, the key of the car. The reader reads the ID-1 in the tag and sends it to the smart phone. Then the user begins a search in the mobile device for the objects with affinities with ID-1 stored, receiving a list with the ID of those objects (ID-2). One of those objects will be the car in this example, so the user can ask for a service that shows the Last Known Location (core service) with each related object ID-2. After receiving the centroids for each interaction with related objects, the smart phone can show the locations of those interactions superimposed over a map. The user will be able to identify his car with this information, or will be able to visit it location shown to find his car.

### 3.2. Affinity Relationship Example

It is a common situation that we buy a home appliance, put away its documentation (user manuals and warranties) and quickly forget it. The appliance will eventually break and we will need (1) to recover this documentation and (2) to know if the warranty period has expired.

**Figure 5.**
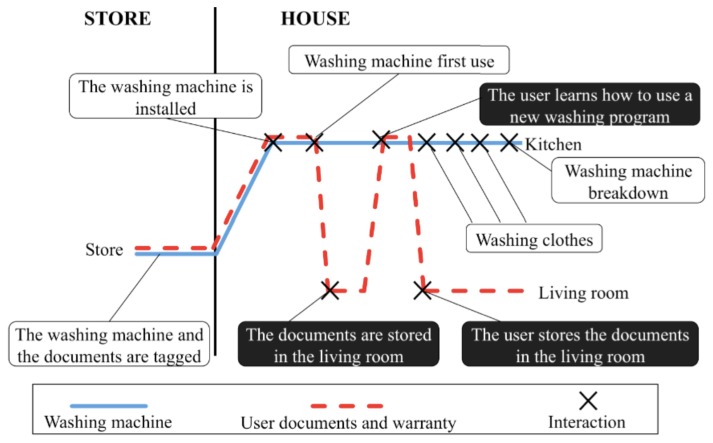
Lifeline of the Washing machine and its documentation.

Dig-Mem enables a record of our interactions with the home appliance and its documentation. Figure 5 shows the lifelines of the washing machine (showed with a solid line) and its documentation (showed with dashed line). The interactions between the things and the Dig-Mem are marked with a cross. The left part of Figure 5 represents the period of time while the product is in the store. The right part of Figure 5 shows the time period in which the washing machine, and its documents, is in the user house. The different interaction points are described with a short text with white background for the washing machine interactions and with black background for the documents interactions.

Figure 5 shows when the date of arrival of the appliance at home was recorded (our first interaction with the thing). The difference between that date and the current date, will produce the needed information about the warranty expiration. We can also observe that the washing machine and the documentation stay together while the appliance is in the store but when it arrives at the user house their locations split up. While the washing machine is installed in the kitchen the documents are stored in other room; the user Dig-Mem will record both locations. The documents location is going to change each time the user gets them to learn how to use a new washing program. The user Dig-Mem also remember each time the user utilizes the washing machine.

If the washing machine breaks down the Dig-Mem will have stored the data the user will need in order to find the washing machine documents and to know if the warranty period has expired or not. Furthermore, we can know the location of the documentation using spatial affinity between them. Whenever we touched the documentation, mostly it was to learn the use of the washing machine (see Figure 5), so both things will appear in Dig-Mem with a spatil affinity (compared for example with the temporal affinity between the washing machine and the clothes).

## 4. Aggregating Interactions into Activities

By means of the interactions and the affinities, we can recognize when something is touched and when two objects share the same space or time frame when are used. However, we need a bigger entity to model more complex actions like driving or having dinner, to do so we define the activities. For constructing our activities we use and Emerging Pattern based recognizer.

Emerging patterns (EP) is a new type of knowledge pattern that describes significant changes (differences or trends) between two classes of data [18]. An EP is a set of items whose frequency changes significantly from one dataset to another. Like other patterns or rules composed of conjunctive combinations of elements, EPs can be easily understood and used directly.

Suppose that a dataset D consists of many instances. An instance contains a set of items *i.e.*, an itemset), where an item is an attribute-value pair. The support of an itemset *X*, *supp_D_*, is *count_D_*(*X*)/|*D*|, where *count_D_*(*X*) is the number of instances in *D* containing *X*. A pattern is frequent if its support is no less than a predefined minimum support threshold. Unlike frequent patterns, EPs are concerned with two classes of data.

### Definition 1

*Given two different classes of datasets D_1_ and D_2_, the growth rate of an itemset X from D_1_ to D_2_is defined as:*

(1) $$\text{GrowthRate}(X)=\{\begin{array}{cccc} 0, & {\text{if supp}}_{1}(X)=0 & \text{and} & {\text{supp}}_{2}(X)=0\\ \infty , & {\text{if supp}}_{1}(X)=0 & \text{and} & {\text{supp}}_{2}(X)>0\\ \frac{{\text{supp}}_{2}(X)}{{\text{supp}}_{1}(X)}, & \text{otherwise} & & \end{array}$$

EPs are those itemsets with large growth rates from *D*_1_ to *D*_2_.

### Definition 2

*Given a growth rate threshold ρ > 1, an itemset X is said to be an ρ — Emerging Pattern (or simply EP) from D_1_ to D_2_ if GrowthRate(X) ≥ ρ*.

We have applied EPs in a similar way than in [9]. Each observation performed with our RFID-watch contains an RFID tag, a time mark and a human-readable location. The time mark stored enables us to compare the time of day where each instance occurs (*i.e.*, at 8:00 in the morning) therefore we can distinguish among activities that share objects and locations but occur at different time frames (*i.e.*, having breakfast, lunch or dinner). In order to apply EPs we have two different phases, the training phase and the activity recognition phase. In the training phase, EPs are extracted from the training dataset, while in the activity recognition phase, previously extracted EPs are applied to recognize activities from the daily dataset. We are aware that not all people do the activities in the same way, thus this may affect the accuracy of activity recognition. In this paper, we apply the techniques used by [9]. However, the accuracy of activity recognition is out of scope of this work.

In order to “train” the system, we need to select the activities set that we want to recognize and use them as raw data for the training phase. To select the activitie we identified the Activities of Daily Living (ADLs) and the Normal Daily Activities (NDAs) from [19]. We select a subset of that activities and perform them several times while using our RFID-watch to create the training Dataset *D_t_*. Then we compare the subset of the dataset of each activity (*D_i_*) with the reference dataset for that activity *DR_i_* (whole dataset *D_t_* minus activity dataset *D_i_*). We extract the EPs from those two datasets (*D_i_*, *DR_i_*) applying the algorithm presented in [20].

Table 1 shows and example of the measurements registered with our RFID-watch during the execution of brushing teeth activity. This example activity has been shortened (brush the teeth in 45 s instead of 3 min) in order to better illustrate the EPs application.

The observations are then grouped into 15 s groups, the activities recognized by our system last enough time to be recognized in frames of 15 s. In the transition between one activity and the next, both activities will be mixed, but they last enough time so that mixed data has low weight in the extracted EPs. Table 2 shows the aggregation of the brushing teeth activity observations into 15 s groups.

Finally we apply the algorithm [20] to extract the EPs comparing the activity dataset *D_i_* with the reference dataset for that activity *DR_i_*, Table 3 shows an example of the EPs extracted for the brushing teeth activity.

We are aware that the technique applied for extracting the EPs and recognizing the activities can be improved, but we don't focus on this area, and therefore we don't have any claim on this. There are several works, such as [9,10,18], which are focused on extracting EPs or recognizing activities [8,11,21]. Our objectives are not to extract EPs or recognize activities; we focus on the feasibility of using recognized activities as cues to remember past events by means of the associative thinking of the human mind.

## 5. Memory-Aware Services

We have implemented several services that use the information stored in Digital Memory to test the feasibility of our approach. Core services are built around the dimensions associated with the interactions and with the affinities between them. Domain Specific Services give solutions to domain specific problems by the combination of core services.

### 5.1. Core Services

Core Services are built around the basic dimensions stored in Interactions and the concepts of Affinity between DOIs and Activities. Given the basic dimensions of space and time of interaction of person with things as depicted in Figure 1, several core services can be implemented. By using the temporal dimension, we can locate the first or the last interactions with the thing of interest. The **last known location** core service searches for the interaction with highest timestamp (time of the last interaction) of the user with the thing of interest that she is holding while launching the service. The **first known location** core service searches for the interaction with lowest timestamp (time of the first interaction) of the user with the thing of interest. One example of use of the last known location service could be remembering the location of a book to know where it was placed. The service can show the location of the last interaction between the user and the book.

The affinity between DOIs can also be used to define other kinds of core services. We have implemented a **Lost and Found** (LaF) service, which takes advantage of both temporal affinity and spatial affinity. The service searches Dig-Mem for affinities between this and other DOIs. Because the number of matches can be big due to random temporal or spatial matches between non related things, the service only looks for the objects with highest affinity. For each related object detected, the service offers to the user the time and location of the last interactions between those things. As the name of the service implies, the user can use the service to find lost things by starting it while holding some other thing that the user identifies as having a strong relationship with each other.

The **protocol services** addresses the problem of situations when some activities are done in conjunction and with some order between them, following a protocol. There are one type where we define pre requisites of an activity in terms of the realization, or not, of another activity before. The other type is the post requisites, where one activity must be followed by another one. In both cases we define a threshold time and the service will warn if we do not follow the protocol established.

For example in the kitchen, there is an activity “Turn-on the cooker hood” and another one called “Cook”. The activity “Turn-on the cooker hood” must be done prior to the activity “Cook”, so the user defines an activity protocol service to warn if the second activity starts before the first one is completed. For the time threshold about five minutes should be enough to launch the alarm.

The **goal services** are focused in situations when the user wants to control the amount of repetitions of a particular activity. To do so the user defines some kind of temporality over a particular activity. The user can establish frequencies, like once a day, two times a week or more specific periods like each 8 h. The service can be configured to warn the user with different grades of severity depending on how crucial is the control of the activity. The service will warn if we miss this frequency by defect or by excess. The selection of the frequency will influence also in the notifications from the Dig-Mem, for example we can select 3 times a day or each 8 h and looks the same, but the Dig-Mem will wait until all the period is consumed prior to warning the user. For example if you want to be reminded to take a pill is better to select each 8 h so the Dig-Mem warns you after 9 h without taking the pill, instead of warning at the end of the day that you haven't take any pill.

### 5.2. Domain Specific Services

By combining different core services we can design and implement services that solve domain specific problems. These *domain specific services* offers the user a solution tailored to solve queries that could be hard to express by simply using the core services described above.

In a Library environment where each bookshelf and book are tagged, one domain specific service that can be offered is the search and location of misplaced or lost books. By touching the bookshelf, the service can identify all things with high spatial affinity with it. From those things, the service can eliminate all DOIs with the same last known location as the location of the bookshelf, keeping only the affinities with those object located at other places.

## 6. Dig-Mem Browser

At the time of retrieving the information stored in the Dig-Mem, the need of resolved objects gains relevance. We need to ease the task of recognizing an object only by its DOI. We allow the user to directly input a name for a DOI, but there are other techniques to address this problem.

We have developed a technique called **Tag sharing** for tagged environments, where there are objects shared among different users, for example at home the living room will be the same for all the family. One member of the family could take advantage of the names of objects resolved by the rest of the family. This feature is maximized in environments with a lot of users (more possibilities of finding tagged objects) or where the users share common goals (at work the activities performed with shared objects will similar for more users)

**Figure 6.**
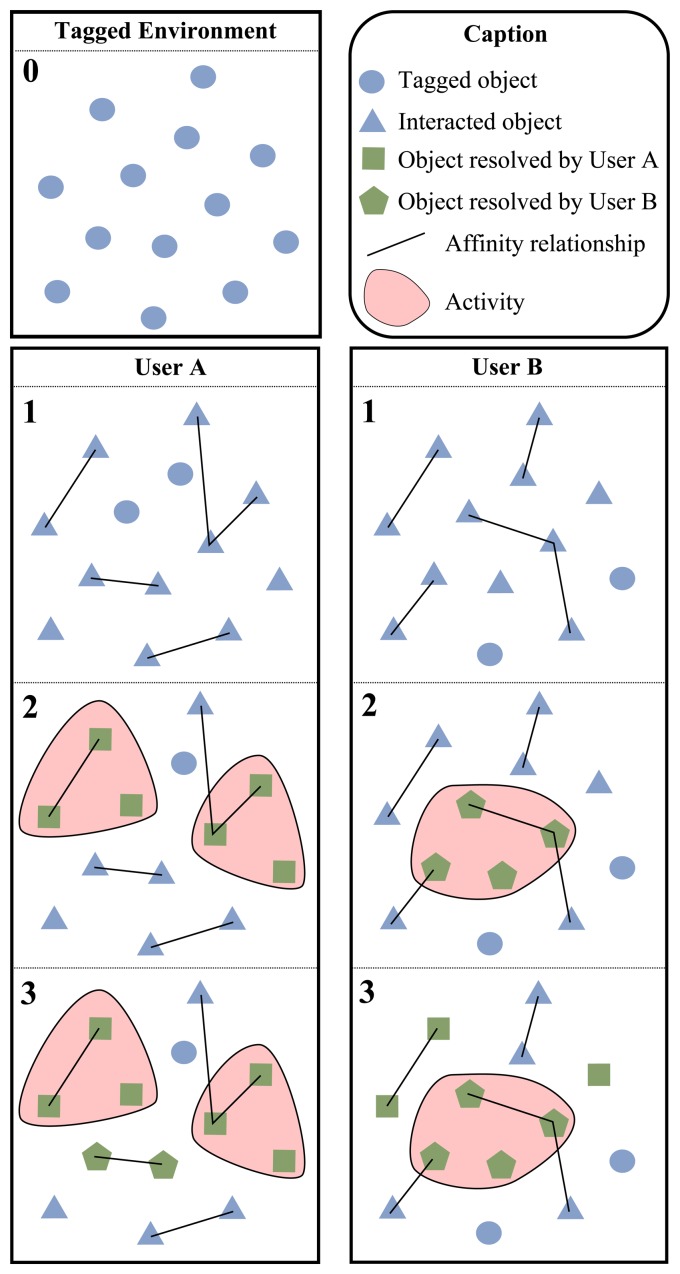
Tag sharing process example.

There is an example of this sharing process in the Figure 6. We have a tagged environment shared by two different users (see block 0 of Figure 6).

Each blue circle represents a tagged object. Users start using the Dig-Mem and, after some interactions, affinity relationships are established among the objects (see block 1). Blue triangles represents objects that the user has interacted with, lines represents affinities among objects. We can see that interactions and affinities are not the same for both users. Then (see block 2) User A has declared two activities, indicated by red shapes, involving some of these objects. As part of the activity declaration process he has resolved the names of the objects with its DOI, now these objects are represented by green squares. To do so, the user is aided by an assistant included into the smartphone. The user can resolve a name for a DOI whenever he wants, by means of the browser. Furthermore, when the user interacts several times with a non-resolved object the assistant will ask for a name. User B has declared only one activity and his resolved objects are represented by green pentagons. At this moment the names used by the users for those specific objects are uploaded anonymously to a cloud. The next time that users interact with a non-resolved object, the name used by other users will be proposed. Finally (see block 3) we can see two objects, represented by green pentagons, have been resolved for User A using the names of user B. As for User B, he has three objects, represented by green squares, resolved thanks to the names of User A.

The task of resolving the names of the DOIs is very important to provide a human readable name of an object to the user in the browser. This task should be performed by the user of our approach. In an evaluation environment, the participants know why is important that they perform this task. However in a free living environment, users may not know the importance of the task, or perhaps, they find it as a cumbersome task. Hence, we need to motivate users to resolve the names of the DOIs. There are some works that are focused on the users' motivation necessary for participation [22].

The Dig-Mem Browser features some views so that users can navigate through the memories of his Dig-Mem. For example there is a view to consult the **memories of a time frame**. The view shows a list of interactions and activities performed in the time frame defined. It can be used as an scheduler that shows all the things done in a day or in a week and has options to hide interactions with non-resolved objects or to show activities that should be performed in the future (because we have defined them as a goal service).

Another example is the **most useful objects view**. This view enables a list of the most interacted objects, giving them a list of the things that are more commonly used by them. But not only the objects, the user can also consult the list of most performed activities. The view can be configured to calculate this only for a particular period of time, for example a weekend or during the holidays. This can be useful for instance to know the things that are essential for the holidays and carry them on the luggage. To compose this list we just used a core service to find the objects with more interactions in the Digital Memory.

The view **traveler objects** shows the user a list with the objects that he usually carries on himself. These objects are essential for the user as he always have them wherever he goes, and can be important to the user to be sure that he is carrying all of them. To elaborate this list we search in the Digital Memory for the objects that appear in the maximum number of locations possible.

## 7. Evaluation and Results

The services described in this paper have been tested by university staff and third year undergraduate students. The number of people who were involved in the evaluation was twelve. In order to know the experiences (positive and negative) of the participants of the university staff, we did a personal interview at the end of the evaluation. In the case of the students, the evaluations were a mandatory assignment for their Human-Machine Interaction course. They had to do a final paper for this subject describing their usability assessment, and adding their thoughts about the whole activity, with suggestions for new Domain Specific Services and possible improvements.

The system for background feed was built using Open-Source Electronic Arduino prototype platform which reduced considerably the time and expertise needed to build the wristwatch. We used the same smartphone, a Nexus S for foreground feed testing, but in background feed tests its NFC reader was turned off. We put a flag in every Dig-Mem record to distinguish between foreground and background feed, but we used all records for every evaluations.

**Figure 7.**
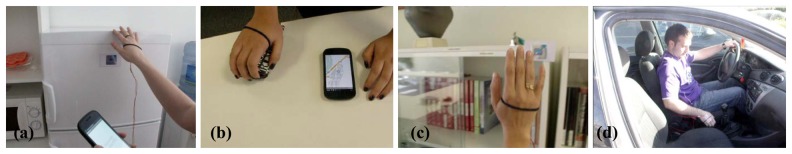
Examples of students using their digital memories in several environments: (**a**) RFID tags were attached to some kitchen appliances. The concierge can know the warranty expiration date of the damaged refrigerator; (**b**) Student is touching her wallet to find her lost credit card by using the LaF service; (**c**) Librarian is avoiding to misplace books by touching the book first and then the bookshelf by means of a domain specific service for the library; (**d**) User is parking his car and his present location is stored in digital memory by means of background feed (the RFID tag was attached to the handbrake).

Figure 7 shows some of our students using the services described in this work:

In the next section we will emphasize some of the findings we have obtained through the analysis of the student behaviour while using the memory-aware services detailed in this article.

### 7.1. Comparing Background and Foreground Memory Feeds

We gave the students freedom for populating their Dig-Mem using both the wristwatch prototype and the RFID reader integrated on the smartphone, in order to study which technique is more successful for populating Digital Memory.

*Background feed technique* turned out to achieve a higher number of stored interactions points than *foreground feed technique* because students were not required to think about interactions with things in their daily tasks. According to student's experiences, a lot of them stopped touching objects with the mobilephone because they forgot they were meant to do it or it was uncomfortable for them to use the mobilephone this way because it was sitting on their pocket. They were aware of the need to touch enough things with their smartphone when they tried to use memory-aware services and these services were not able to recover useful information.

### 7.2. Tag Deployment by Users

We explained to the students the basic working of RFID tags and supplied each student with a small number of tags to be deployed at their homes and a Dig-Mem device. Our intentions were: (1) to evaluate the capability of a technological user to take advantage of the Dig-Mem; (2) to discover domain specific services for home applications; (3) test the idea and find problems or improvements. Students were expected to deliver a paper describing their usability assessment of the processes of populating the Dig-Mem, creating activities, using services at the smartphone and browsing through the Dig-Mem. They were also expected to supply their thoughts about the whole activity, with suggestions for new Domain Specific Services and possible improvements.

Students report some problems during the installation and reading of RFID tags. Some metallic surfaces interfere with the RFID tag and makes them impossible to read. We knew that there are newer RFID tags that are optimized for placement near liquids and metal, but we couldn't get this type of RFID tags before performing the evaluation. A student also has a problem with the security system of their car, that was altered with the presence of the RFID tag.

Some students reported that they forgot to put on the device some days, they weren't accustomed to wear a watch, so for them was a hassle to wear the device. Some other users that usually wear a watch, reported some inconvenience wearing the device. This problems were blamed on the lack of aesthetic value and big size of the prototype. Despite of this, we created a device encapsulated into a glove, to be used in industrial environments, and validating the idea that the device could have a different encapsulation (like an elegant bracelet).

With this experience we received valuable feedback from our students on the kind of services to offer in this context and the things to tag, so we began to write a possible catalog of services useful to a set of users. For example, locating the audio-video remote or remembering the activities of pets. However, due to the heterogeneity of proposals from our students, we were forced to conclude that in environments as particular as home, we should explore end-user programming techniques [23] to involve the user even more in the design and constructions of services for this very specific and personal domains.

## 8. Dig-Mem *vs.* Natural Memory Evolution Case Study

We have performed a case study to compare both Dig-Mem and natural memory evolution throughout time. In particular we want to evaluate the ease of remembering things performed in a regular day.

We recorded the case study to compare if the memories that we are going to collect are correct or not. Because of that we used a closed area, a laboratory, place of work of some internship from Universidad San Jorge. We decided to use this environment due to the kind of interactions that take place there. In a classroom the students mainly listen to the lecturer and there is much less interaction with the environment, by contrast in a laboratory of interns the tasks performed are linked to the objects present in that environment, and involve less listening and more object manipulation.

We have chosen interns for the case study so they are familiar with the environment. We choose four interns, all of them are part of a research group. The group of interns is not related to the Dig-Mem project, they don't know about the device before the case study, but they have technical background, avoiding problems with the operation of the device.

The case study starts explaining the interns the use of the device; we give one device for each intern and tag the laboratory with their help. We encourage the interns to tag also personal objects used at the laboratory as their wallets or cellphones. We place a camera and tell the interns that we are going to record the case study, but we don't specify the objective of the case study, to avoid extra efforts by the interns to remember specific things. They know they are going to participate in a case study, they also know they are going to be recorded by a camera and which objects are tagged, but they don't know that the goal is to compare the Dig-Mem and natural memory.

The second day recorded by the camera (The first day interns were excited about the case study and the camera) will be the reference day (hereinafter **ref-day**), and will be used to evaluate their memories as time passes. It is important to notice that the Dig-Mem will be then locked to start after that day, by this we avoid that the interns remember the things done that day consulting the Dig-Mem.

At the end of the day we met the interns and told them that we need to evaluate their memory. We want to minimize the effect of our questions over their natural memory, so instead of asking directly about if some actions where done or not, we ask them to tell us everything that they have done during the ref-day. We emphasized the importance of details and ask them to take as much time as they need because the intern that remembers more correct things will win a prize. We don't give them feedback, so they don't know how well have performed the test.

We perform this activity three times more, following the same pattern. It is important that the interns don't know if there is going to be another meeting, so they don't make extra efforts trying to remember the actions of the day (or even cheating by writing things down).

**Figure 8.**
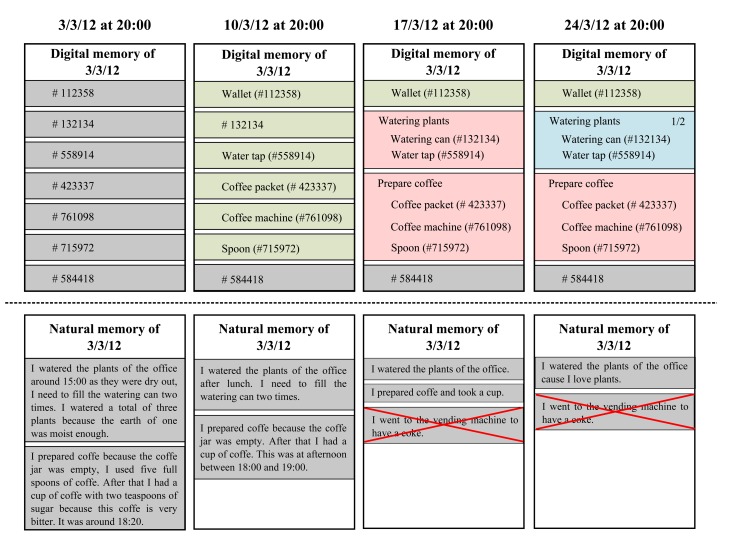
Comparison between Dig-Mem (top) and Natural Memory (bottom). Representation of a subset of data collected during the case study from the devices and the written stories written by the participants.

After the last meeting we conclude the phase of data collection of the case study. The Figure 8 is a representation of a subset of the data collected from the devices and the written stories written by the interns. We only show the information of one intern to simplify the image, but the results where similar for all the interns.

In the first meeting, the Dig-Mem is just a set of interactions. There are no objects resolved so we can't figure which element represents each DOI. These memories can't be interpreted only looking at the Browser (although we can use them trough core services). By contrast, natural memory has plenty of details. We can watch how the intern remembers that he has watered the plants, but also details as the number of times they filled the watering can or why they decided to perform the action. With the coffee we observe the same; it offers plenty of details, such as the number of spoons and even personal assessments about the action.

The first row shows the information extracted from the Dig-Mem in the meetings. The second row shows the information extracted from the written stories of the interns, there is shown only the data relevant to the activities we are showing, not the full report. Each of the columns shows one of the meetings results, the first meeting is most left.

For the second meeting, the interns have started using the Dig-Mem and they have resolved some objects; when we consult the memories of the ref-day they make more sense, as we can confirm some objects that the intern interacted with. We don't know exactly what happened but is sure that the intern touched the coffee machine or the water tap. By contrast the memories of the natural memory are less detailed than in the first meeting. These activities are still remembered, but some details are forgotten. In addition, the times are fuzzier.

For the third meeting we observe a big change in the two memories. The interns have defined activities of interest for them, when we browse the memories of the ref-day we observe how the activities have changed the interpretation of the memories. We observe that the intern watered the plants and prepared coffee. By contrast the natural memory has been reduced to just the activity; there are no details of them. In addition the interns started to mix memories of different days. They attributed things done other days in that week (we check that with the record) to the ref-day, in the image we can see a representation of that.

For the last meeting we observe that the interns have started to use the Digital Memory to control their activities. For instance they have defined a goal service over the activity watering plants to be sure of performing the activity two times a week. When we observe the memories of the ref-day, the activity watering plants is interpreted as the first of the two planned for the week. By contrast the natural memory has lost even more information, and some of the mixed memories are repeated. We can observe how some of the tasks are even totally forgotten, like preparing coffee.

In summary, the interpretation of the Dig-Mem has evolved over the time, making the memories recorded in the ref-day understandable and giving them some value for the user as time passes. The hours of the Dig-Mem are very accurate. By contrast the natural memory was plenty of details at the beginning, giving much more information than the Dig-Mem (even the one of the last meeting). As time passes this natural memories have suffer from degradation, up to the point of mixing memories of different days or totally forgetting whole activities.

At the end of the case study we unlocked the data of the ref-day of the Digital memories (that has been locked throughout all the case study) and then we showed the browser in that day to the interns. There were different reactions when they saw that information.

- Dig-Mem worked as a stimulus in some cases. When users saw the Dig-Mem, they recall the moment in their memories and contribute new details to that activity.
- Some of the activities where easily recognized by the interns, but for other don't. There are factor that affects the odds of an activity of being remembered
- For a couple of mixed memories the interns didn't recognize the mistake; they assured that device had an error (until they saw the incongruences with the first day and with the record).

It turns out that digital memories not only evolve getting close to the user perception, they also feedback the natural memory. We are currently planning an specific case study to study the influence of the Dig-Mem over the natural memory and detect if there is any kind of synergy when we use both digital and natural memory.

## 9. Related Work

Several groups [24,25] have recently proposed to incorporate humans carrying smart phones in a sensing data collecting loop. Such a novel approach is shortly called **Participatory Sensing.** In participatory sensing, a large number of users carrying smart phones contribute to monitoring the environments with their sensing measurements (for instance, Mobile Millennium [26], Simple Context [27], Urban Atmosphere [28]). That is, users cooperate to sense data that enables services of interest for the collective of users. For example, Participatory Sensing enables generic services such as traffic information [29] or pollution [30] status in their city. However, currently existing deployments have suffered from insufficient participants because participants who voluntarily submit their sensing data found no interest to remain actively in the system without being rewarded [22].

Conversely, memory-aware services require users to individually sense data in order to achieve personal and important services for each individual. In fact, memory-aware services are so personal that if two users switch their digital memories then the resulting services become useless. According to the experimentation with our students, the return of investment (battery power consumption and wearing the wristwatch) is greater than their expectations because they could not find services to complement their lack of memory elsewhere.

Significant amount of research and development is being directed also on monitoring activities of daily living of senior citizens who live alone as well as those affected with certain disorders such as Alzheimer's. This field of research is known as **Embedded Assessment Technology** [31]. Sensors embedded in the home can monitor how older adults interact with objects around the home and can provide objective accounts of behaviors to support self-awareness. Hayes *et al.* [32] monitor the pill-taking task, they developed a smart pillbox that could monitor when a door was opened and how the box was manipulated. They augmented an existing off-the-shelf pillbox with snap action switches to know which doors were open. Lee *et al.* [33] extended Hayes' work by introducing more sensors in the home environment in order to run experimentation that validates the benefits of embedded assessment data. Gartenberg *et al.* [34] describe a mobile health application that collects data relevant to the treatment of insomnia and other sleep-related problems. The application is based on the principles from neuroergonomics, which emphasizes assessment of the brain's alertness system in everyday, naturalistic environments, and ubiquitous computing. However, the focus of the former works is on monitoring rather than compensation as our Dig-Mem does.

On the other hand, the **Instrumented Environments** capture as many kinds of data as possible through sensors or networks, as in MIT's PlaceLab (http://architecture.mit.edu/house_n/placelab.html). There are also other works that perform this type of capture. Liu *et al.* [35] and Nemmaluri *et al.* [36] use a RDIF localization technique that uses steerable antennas to “sweep” a room, discovering, localizing and indexing tagged objects. None of these works say anything about using their approach to improve memories.

**Lifelogging Technologies** [37] have the potential to provide memory cues for people who struggle with episodic memory impairment. In particular Lee *et al.* [4] propose to equip memory-problem patients with a device for taking random pictures daily. Then, such pictures are used to improve memory skills by questioning the patient to remember about what he was doing at those moments. Doherty *et al.* [38] share their experiences in designing computer-based solutions to assist people review their visual lifelogs and address this contrast. These approaches are not focused on directly compensating effects of memory loss. Instead, the proposal intends to assist the patient in a treatment for rehabilitating his memory.

Finally, **Specific Location Services** [39,40] distribute an object search query to a subset of mobile users that are likely to find a given item. That is, object owners can search for their objects using the infrastructure of mobile phones carried by other users. The successful of this approach relies on environment conditions such as the participant density and their mobility. In our work, the location services (which are based on the previous interactions of the user) are just part of the core services that we use to provide the domain specific services. Our work does not focus on location services; our main goal is to build up a Dig-Mem that provides memory-aware services. These services are influenced by the current dimensions of the memory (DOI, time and space) but by means of experimentation, we are introducing more dimensions to enrich the Dig-Mem and therefore both the memory-aware services and the Dig-Mem browser.

## 10. Conclusions

With more and more RFID tags being added to our surroundings Memory-Aware Services can play an important role in our lives providing us an important complement to our fading memory. Specifically, Memory-Aware Services can answer generic questions such as where are my keys and even more complex problems such as if I have followed correctly my medicine prescriptions or if I have watered the plants enough times this week.

This paper shows how Dig-Mem provide services based on our past interactions with things. We can trigger these services either by touching a physical thing (and digitally remembering our interactions or activities with this thing) or even without having access to the physical object by means of its affinity relationships with other things that we can touch.

In this work, we have developed a device to support the approach enabling the user for populating the Dig-Mem. The digital memories are stored in terms of interactions, affinities, activities, goals and protocols. We have also developed a Dig-Mem Browser to enable users themselves to navigate through their digital memories. Furthermore, this Dig-Mem Browser also takes advantage of the developed Tag Sharing technique to speed up DOIs resolution.

This approach has been successfully validated in different environments at a Technological Park by our students. Besides, we have obtained some interesting findings about the capability of a technological user to take advantage of the Dig-Mem and create a tagged environment.

We have observed the evolution of the Dig-Mem over the time noticing a improvement in the presentation of the memories as time passes and the device is used, compared to natural memories, where the level of detail decreases over time. This has lead us to possible synergies between digital and natural memories.

## Figures and Tables

**Table 1.** Measurement registered with our RFID-watch during the execution of the brushing teeth activity.

**RFID**	**Timestamp**	**Location**
Toothbrush	9:00:00	Bathroom
Toothbrush	9:00:02	Bathroom
Toothpaste	9:00:05	Bathroom
Water	9:00:10	Bathroom
Water	9:00:15	Bathroom
Toothbrush	9:00:17	Bathroom
Toothbrush	9:00:25	Bathroom
Toothbrush	9:00:27	Bathroom
Water	9:00:30	Bathroom
Toothbrush	9:00:35	Bathroom
Water	9:00:37	Bathroom
Toothpaste	9:00:40	Bathroom
Toothbrush	9:00:45	Bathroom

**Table 2.** The aggregation of the brushing teeth activity observations into 15 s group.

**RFID**	**Timestamp**	**Location**
Toothbrush, Toothpaste, Water	9:00:00–9:00:15	Bathroom
Toothbrush, Water	9:00:15–9:00:30	Bathroom
Toothbrush, Toothpaste, Water	9:00:30–9:00:45	Bathroom

**Table 3.** Emerging Patterns extracted for the brushing teeth activity.

**EP**	**Support in D_i_**	**Support in DR_i_**	**Growth Rate**
Toothbrush, Toothpaste, Water, Bathroom, 9:00:00	66%	0%	∞
Toothbrush, Water, Bathroom, 9:00:00	100%	5%	20
Toothpaste, Water, Bathroom, 9:00:00	66%	5%	13
Toothbrush, Toothpaste, Bathroom, 9:00:00	66%	3%	22
Toothbrush, Bathroom, 9:00:00	100%	10%	10
Water, Bathroom, 9:00:00	100%	30%	3
Toothpaste, Bathroom, 9:00:00	66%	10%	7
